# MeCP2 Related Studies Benefit from the Use of CD1 as Genetic Background

**DOI:** 10.1371/journal.pone.0153473

**Published:** 2016-04-20

**Authors:** Clementina Cobolli Gigli, Linda Scaramuzza, Anna Gandaglia, Elisa Bellini, Marina Gabaglio, Daniela Parolaro, Charlotte Kilstrup-Nielsen, Nicoletta Landsberger, Francesco Bedogni

**Affiliations:** 1 San Raffaele Rett Research Unit, Division of Neuroscience, San Raffaele Scientific Institute, Milan, Italy; 2 Institute of Surgical Pathology, University Hospital Zurich, Zurich, Switzerland; 3 Department of Biotechnology and Life Sciences, University of Insubria, Busto Arsizio, Italy; 4 ZardiGori Foundation, Milan, Italy; 5 Department of Medical Biotechnology and Translational Medicine, University of Milan, L.I.T.A., Segrate, Italy; Institute of Genetics and Biophysics, ITALY

## Abstract

*MECP2* mutations cause a number of neurological disorders of which Rett syndrome (RTT) represents the most thoroughly analysed condition. Many *Mecp2* mouse models have been generated through the years; their validity is demonstrated by the presence of a broad spectrum of phenotypes largely mimicking those manifested by RTT patients. These mouse models, between which the C57BL/6 *Mecp2*^*tm1*.*1Bird*^ strain probably represents the most used, enabled to disclose much of the roles of Mecp2. However, small litters with little viability and poor maternal care hamper the maintenance of the colony, thus limiting research on such animals. For this reason, past studies often used *Mecp2* mouse models on mixed genetic backgrounds, thus opening questions on whether modifier genes could be responsible for at least part of the described effects. To verify this possibility, and facilitate the maintenance of the Mecp2 colony, we transferred the *Mecp2*^*tm1*.*1Bird*^ allele on the stronger CD1 background. The CD1 strain is easier to maintain and largely recapitulates the phenotypes already described in *Mecp2*-null mice. We believe that this mouse model will foster the research on RTT.

## Introduction

Mutations in the X-linked *MECP2* gene (methyl-CpG-binding protein 2) cause a large spectrum of neurological disorders affecting almost 1 of 4000 individuals worldwide. Rett syndrome (RTT) was the first identified and certainly the most thoroughly characterized *MECP2*-related disease [1,2]. RTT is a devastating genetic disorder that affects primarily young girls and represents the most common genetic cause of severe intellectual disability in females. RTT is usually characterized by a severe regression phase occurring between 12 and 18 months of age that leads to the loss of most, if not all, the previously acquired skills. Clinical symptoms include impaired cognitive and motor abilities, communication dysfunctions, breathing abnormalities, seizures, social withdrawal, hypotonia, scoliosis and stereotypic hand movements. So far, more than 500 different RTT-causing mutations have been identified, including missense, nonsense, frameshift mutations and deletions (catalogued at http://mecp2.chw.edu.au). It is generally assumed that these mutations cause the loss of Mecp2 functions [3].

The generation of several mouse models carrying different *Mecp2* mutations has been instrumental to improve our comprehension of MeCP2 roles and the mechanisms underlying *MECP2*-opathies [4,5]. Transgenic animals have also demonstrated that RTT like features in mice are not irreversible and that part of these defects can be rescued even at late stages of disease progression [6]. Such results, confirmed by several other studies [7–9], have further boosted the necessity of using *Mecp2* mouse models both for basic and preclinical studies.

Currently, three RTT mouse models are predominantly used. Two of them, both generated in 2001, are *Mecp2*-null animals [10,11]. The laboratory of Adrian Bird produced the *Mecp2*^*tm1*.*1Bird*^ strain on a C57BL/6 background, removing exons 3 and 4 of the *Mecp2* gene, therefore deleting all the coding portion but the first eight N-terminal amino acids [10]. Rudolph Jaenisch and his collaborators developed a null model on a mixed genetic background (129, C57BL/6 and BALB/c) devoid only of exon 3, therefore including most (116 residues) of the Methyl-CpG-Binding Domain of the protein. The presence of Mecp2 peptides smaller than the full-length protein has been described in this animal [11]. A strain expressing a truncating mutation often found in RTT was generated in 2002 (*Mecp2*^308^, [12]). Importantly, all these mice develop symptoms that recapitulate very well many RTT features. In the male progeny of each line, the onset of several neurological impairments follows an apparently normal development typically lasting 3–5 weeks after birth; death occurs 2–4 weeks later. Symptomatic hemizygous males show reduced movements, abnormal gait, hind limb clasping, reduced weight, tremors and poor general conditions [10,11,13,14]. Of note, given *Mecp2*-null male mice are sterile, null females cannot be generated; *Mecp2*^*+/-*^ heterozygous females are therefore necessary to maintain the colony. Females are characterized by a late manifestation of overt symptoms, generally appearing between 3 and 6 months of age [10,11]. Because of the faster appearance of pathological features, the male model has been vastly preferred.

Due to its early commercial availability, the C57BL/6 *Mecp2*^*tm1*.*1Bird*^ strain probably represents the most widely used mouse model of Mecp2 functions and Rett syndrome. This model displays such a wide number of harsh symptoms that it is often quite hard to distinguish between those directly ascribable to the absence of Mecp2 and those that are, instead, consequence of the poor condition of the animal. Interestingly, the null phenotype tends to progressively deteriorate as the colony ages, possibly hardening the ability to directly link specific features to the absence of Mecp2 and not to the general health status of the animal. Accordingly, rearing these animals is quite challenging, especially considering pre-clinical studies that require large cohorts of mice [15]. In fact, heterozygous dams frequently cannibalize their relatively small litters and progressively take less care of their progeny along with colony aging, which can obviously have detrimental effects on the phenotype the progeny will develop. In fact, it is well demonstrated that maternal deprivation in early post-natal development leads to neuroplasticity defects later in adulthood [16,17]. Through the years some housing methods have been suggested to facilitate the management of the colony; for example, it is suggested to supplement cages of pregnant females with irradiated sunflower seeds and face them towards the light source. Moreover, a good practice is to limit stressful cage maintenance procedures both before and after delivery. Eventually, it is preferred to always mate *Mecp2*-heterozygous females with the same male [15]. All these suggestions appear only modestly helpful and are used only by certain groups not on a regular basis. To ameliorate such poor conditions, researchers have often transferred the mutant allele on different genetic backgrounds, such as 129/C57BL6, F1 hybrid (i.e. FVB.129F1; 129.B6F1) or even mixed F1 (129S1/SvImJ females crossed with B6/CBA males) [18–21]. However, the genetic background could affect the phenotype; indeed, only in the C57BL/6 background *Mecp2*-null mice exhibit reduced weight [10, 11]. Verifying whether the defects described in each study could be ascribable to the lack of Mecp2 itself rather than to the possible roles played by different modifier genes would thus be highly informative.

We hypothesized that by transferring the *Mecp2*^*tm1*.*1Bird*^ genetic modification on the outbred CD1 (ICR) genetic background [22], we could both simplify the management of the colony and highlight the influence of the background on some of the RTT like phenotypes. CD1 mice are in fact characterized by a significant robustness and by the ability to produce large litters [22].

Therefore, as mainly instructed by references 6 and 16, we have examined the general breeding properties of the CD1 line and its symptoms over time. Furthermore, we have assessed whether molecular, neuronal and behavioural phenotypes that characterize the *Mecp2*-null mice are maintained in the new genetic background.

## Materials and Methods

### Animals

The *Mecp2*-null mouse strain was originally purchased from Jackson Laboratories (B6.129P2(C)-*Mecp2*^tm1.1Bird^/J); this strain was generated on a C57BL/6 background [10]. We crossed C57BL/6 *Mecp2* heterozygous females with CD1 wt male mice (Crl:CD1(ICR); Charles River) and kept backcrossing each generation of CD1 heterozygous females with new CD1 males. This backcrossing strategy is now well above the 10^th^ generation. All animals were kept in our on site animal room and daily checked for general health conditions. All procedures were performed in accordance with the European Community Council Directive 86/609/EEC for care and use of experimental animals; protocols were approved by the Italian Minister for Scientific Research and by the San Raffaele Scientific Institutional Animal Care and Use Committee in accordance with the Italian law.

### Genotyping

Mouse genotypes were determined through PCR on genomic DNA purified from tail biopsy. Biopsies were obtained within the second and the third week of life. Forward PCR primer sequences: 5’-ACCTAGCCTGCCTGTACTTT for the identification of the null allele, 5’-GACTGAAGTTACAGATGGTTGTG for the wt allele. Common reverse primer sequence: 5’- CCACCCTCCAGTTTGGTTTA. The obtained PCR products were: a single band of 450 base pairs (bp) for the *Mecp2*-null mice; a single band of 400 bp for the wt animals and the two bands for heterozygous females.

### Behavioural analyses

CD1 wt, *Mecp2*-null and *Mecp2*^*+/-*^ heterozygous mice were assayed as described by Guy et al [6]. To avoid any bias, an investigator blind to the genotypes of the tested animals performed all the analyses. Mouse symptoms were scored according to the following scale [6]: 0 if the symptom was absent, 1 if the symptom was present but mild, 2 when the symptom was severe. Mobility, hindlimb clasping, tremor and general condition were the symptoms analysed. To test mobility, mice were allowed to move freely on the bench; the spontaneity of the movement was evaluated. Hindlimb clasping was assessed by holding mice by the tail: a score equal to 1 was given to animals showing clasping for more than 3 seconds and equal to 2 when longer than 10 seconds. Tremor was assessed by placing mice on the palm of a hand. Eventually, the term “general conditions” refers to the assessment of the integrity of the fur, eye crustiness and body posture. To be noticed, all animals that either scored “2” during tremor assessment or rapidly lost weight were euthanized for ethical reasons. At the experimental endpoint, animals were euthanized through cervical dislocation after Avertin (Sigma) deep anaesthesia. At the time of death, the main organs were dissected and weighed.

The cognitive functions were assessed through Novel Object Recognition (NOR) test. The experimental apparatus used for the NOR test was an open-field box, 43x43x32 cm (Ugo Basile, Varese, Italy) made of Plexiglas, placed in a dimly illuminated room. Animals performed each test individually. Briefly, each animal was placed in the arena and allowed to explore two identical, unknown objects for 10 minutes (familiarization phase). 30 minutes (short-term memory) or 24 hours (long-term memory) after the familiarization phase, mice were placed again in the same arena containing a new object that replaced one of the two already tested. During the test phase the time spent exploring the familiar object (Ef) and the new object (En) was recorded separately by two observers blind to the groups and the discrimination index was calculated as follows: [(En-Ef)/(En+Ef)] x 100.

### qPCR

Symptomatic *Mecp2*-null CD1 animals (P60) and *Mecp2*^*+/-*^ heterozygous females (P≥240), and their wt siblings were deeply anesthetized with Avertin (Sigma) and sacrificed through cervical dislocation. Cortices from 11 wt, 9 *Mecp2*-null males, 8 *Mecp2*^*+/-*^ heterozygous and 9 wt females were dissected and total RNA was purified using the RNeasy Mini Kit (QIAGEN), after mechanical trituration of the tissue using a glass-glass potter. RNA was then retro-transcribed using commercial kits (Qiagen). qPCR reactions were run using the following primers: BDNF (Fwd 5’-AAGTCTGCATTACATTCCTCGA, Rev 5’-TTATCAATTCACAATTAAAGCAGCAT), HIF1α (Fwd 5’-CCCAGTGAGAAAAGGGAAAG Rev 5’-ATTTGACGGATGAGGAATGG), Mecp2 (Fwd 5’-AAACCACCTAAGAAGCCCAAAT, Rev 5’-TTGACAAACAAGTTTCCCAGGG), 18S (Fwd 5’-GTAACCCGTTGAACCCCATT, Rev 5’-CCATCCAATCGGTAGTAGCG). RNA levels were normalized to the 18S internal standard and expressed as percentage of the wt (considered as 100%) ± SEM.

### Western blot

Asymptomatic/early symptomatic (P30, n = 4), symptomatic (P60, n = 5) *Mecp2*-null CD1, symptomatic (P≥240, n = 7) *Mecp2*^+/-^ female animals and their wt siblings (n = at least 3) were deeply anesthetized with Avertin (Sigma) and sacrificed. Cortices were dissected and mechanically triturated with a glass-glass potter. Proteins were extracted on ice in lysis buffer (Tris-HCl pH 7,4 50 mM, NaCl 200 mM, Triton 1%, EDTA 2 mM, DTT 1 mM, Protease Inhibitor (Roche) and phosSTOP (Roche)). Samples were then sonicated and centrifuged for 15 min. After centrifugation, the supernatant was collected and proteins quantified (Bicinchoninic Acid Assay, Thermo Scientific). Equal amounts of proteins were loaded on SDS-PAGE and blotted onto nitrocellulose; after blocking, nitrocellulose membranes were incubated overnight with the following primary antibody: anti-S6 ribosomal protein (1:1000, Cell Signaling), anti-phospho S6 ribosomal protein 240–244 (1:1000, Cell Signaling), anti-GAPDH (1:1000, ThermoFisher) and anti-Mecp2 (1:1000, Cell Signaling). After washing, filters were incubated with HRP-conjugated secondary antibody (1:10000, Sigma) and positive signals visualized through chemiluminescence (Thermo Scientific). The obtained films were scanned and analysed using ImageJ. Protein levels were expressed as percentage of the wt (considered as 100%) ± SEM.

### Histological analyses

P60 brains were dissected out, grossly cut in serial 3 mm thick sections and fixed in Carnoy’s solution (70% EtOH, 20% CHCl_3_, 10% Acetic Acid) before inclusion in paraffin. 4 μm thick coronal sections produced by microtome slicing of frontal cortical areas from 6 wt and 4 null animals were used for DAPI staining (Invitrogen). Nuclear size and cell density were measured using ImageJ.

### Blood tests

Blood samples were collected from deeply anaesthetized animals (6 wt and 4 ko) that were soon after euthanized to collect tissues for histological analyses. Blood samples were allowed to coagulate at room temperature and then centrifuged to collect the serum. Samples were stored at -20°C for further analyses. Chemical analyses were performed with the ILab Aries analyser (Instrumentation Laboratory, Werfen group, Milan, Italy). Total serum cholesterol and triglycerides were measured using kits and controls supplied by ILab.

## Results and Discussion

C57BL/6 (from now on abbreviated with B6) heterozygous *Mecp2*^tm1.1Bird^ females were crossed with CD1 wt male mice (Crl:CD1 (ICR); Charles River) and the progeny backcrossed for 10 generations (detailed in Materials and Methods). As expected [10], CD1 *Mecp2*-null males are infertile.

**Fig 1:**
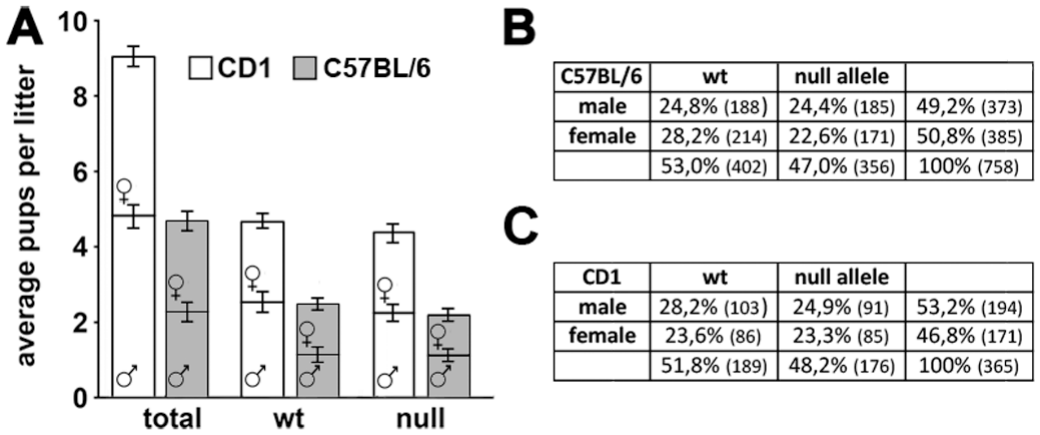
CD1 *Mecp2*^tm1.1Bird-/X^ females have large litters with balanced *Mecp2* alleles. **A**: Litters obtained crossing CD1 *Mecp2*^*-/X*^ heterozygous females and wt males are larger (an average of 9,13±0,39 pups per litter) than those from the same crossing in the B6 background (an average of 4,73±0,17 pups per litter). The percentage of males and females is similar in each litter (roughly 50%) for both the genetic backgrounds. **B**: Although not statistically significant, in the B6 litters the frequency of the null allele results slightly lower than the expected 50%. **C**: The null allele is normally represented in the CD1 progenies. Statistical significance was evaluated through Chi-square analysis, significance is considered when p<0,05.

The first advantage we observed is the duplication of the number of pups per litter (the average pups per litter is roughly five in B6 and nine in CD1), thus obviously increasing the number of null and heterozygous animals obtained from a single litter (Fig 1A). In a time window of one year, the B6 colony exhibited a 33% frequency of cannibalized litters; on the contrary, in the CD1 colony cannibalism resulted only occasional, with an estimated frequency of less than 3%. Moreover, in the majority of the cases, CD1 heterozygous females take better care of their pups, provided that pregnant females are not disturbed immediately before and after delivery.

Jugloff et al. [15] demonstrated that in the B6 colony the number of animals inheriting the null allele (null males and heterozygous females) per litter is under the expected frequency of 50%. Our B6 colony partially reproduces this observation; in fact, we found a reduced, although not statistically significant, number of animals bearing the null allele (*p* = 0,09; Chi square = 2,79 d.f. = 1; Fig 1B). In the CD1 colony the percentage of animals bearing the null allele is roughly 50% and both the heterozygous females and the null males are normally represented (p = 0,5; Chi square = 0,46; d.f. = 1; Fig 1C).

**Fig 2:**
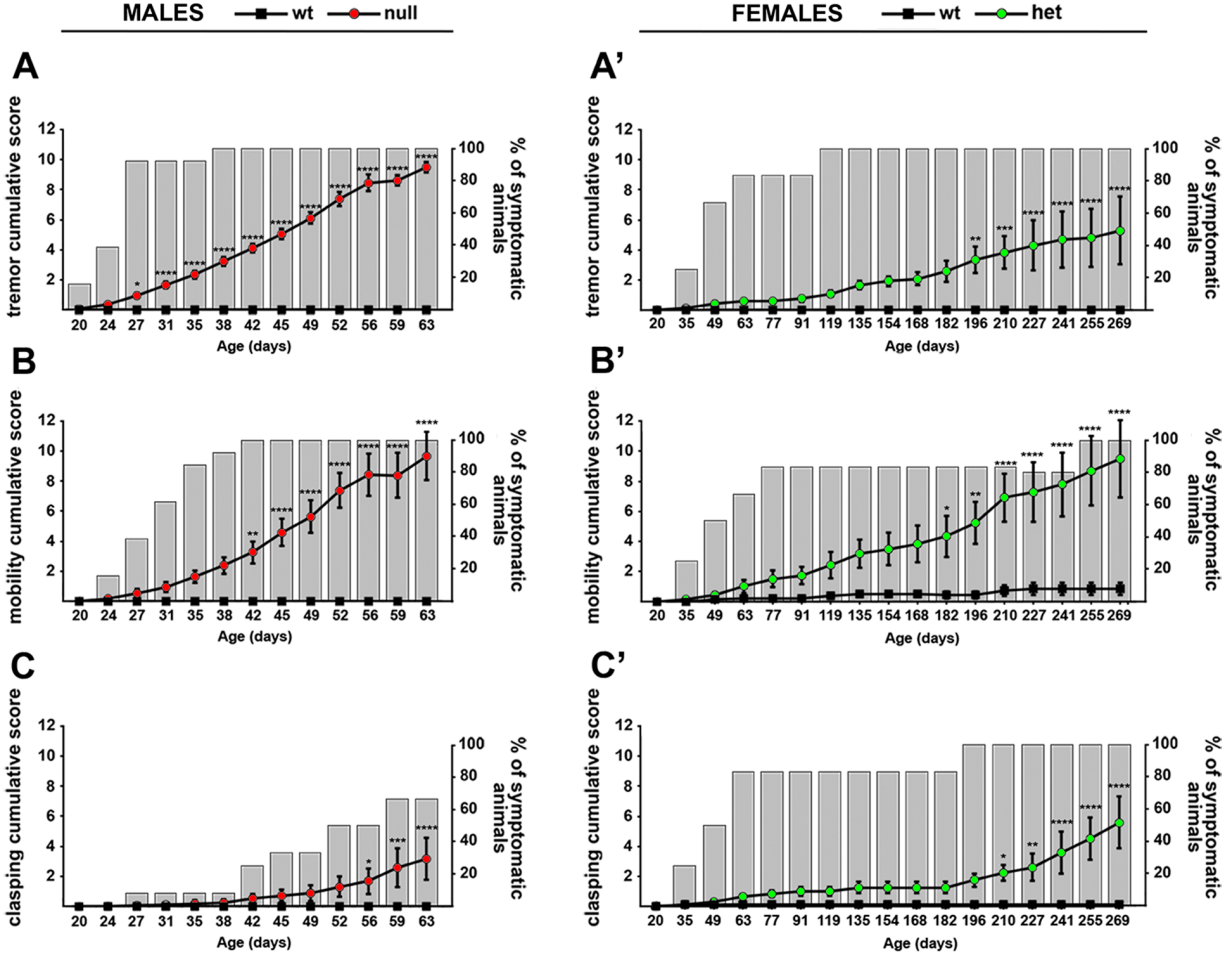
Phenotypic characterization of the CD1 *Mecp2*-null male and *Mecp2*^*+/-*^ female mice. The presence of symptoms (tremor (**A-A’**), mobility (**B-B’**) and clasping (**C-C’**)) was evaluated in null (**A**, **B**, **C**) and heterozygous animals (**A’**, **B’**, **C’**) starting from P20. Continuous lines represent the evolution of the symptomatology, while histograms represent the percentage of null or heterozygous animals displaying the symptom at any given age. Statistical significance was evaluated through two-way ANOVA, Bonferroni’s post-hoc test: * = p<0,05; ** = p<0,01; *** = p<0,001; **** = p<0,0001; n = 15 wt males, 13 *Mecp2*-null, 7 *Mecp2*^*+/-*^ and 7 wt females.

To evaluate if CD1 *Mecp2*-null mice present the same general alterations reported in the B6 null animals, mice were observed every week starting from post-natal day 20 (P20) [6] (Fig 2). Apart from some expected variability, after roughly one month of life, almost all the CD1 null animals showed tremors (Fig 2A). In the same time window, spontaneous movements and mobility begun to diminish and, at 6 weeks of age, all the tested animals displayed mobility impairments (Fig 2B). Hind limb clasping appeared a later and milder phenotype compared to tremors and altered mobility. Indeed, it became evident only at around 7–8 weeks of life and only 60% of CD1 null animals displayed it throughout life (Fig 2C). CD1 heterozygous females show a similar, although shifted in time, progression of phenotypes. Mild symptoms were in fact detected between 3 to 4 months of life. Tremors (Fig 2A’) and mobility impairments (Fig 2B’) were the first to be detected, while hind limb clasping (Fig 2C’) became obvious between 6 and 7 months. It is reported that *Mecp2* mouse models on the B6 background develop symptoms during the same time window (3–8 weeks for males and after 3 months for females), with hind limb clasping and breathing abnormalities following the onset of spontaneous movement issues [10].

**Fig 3:**
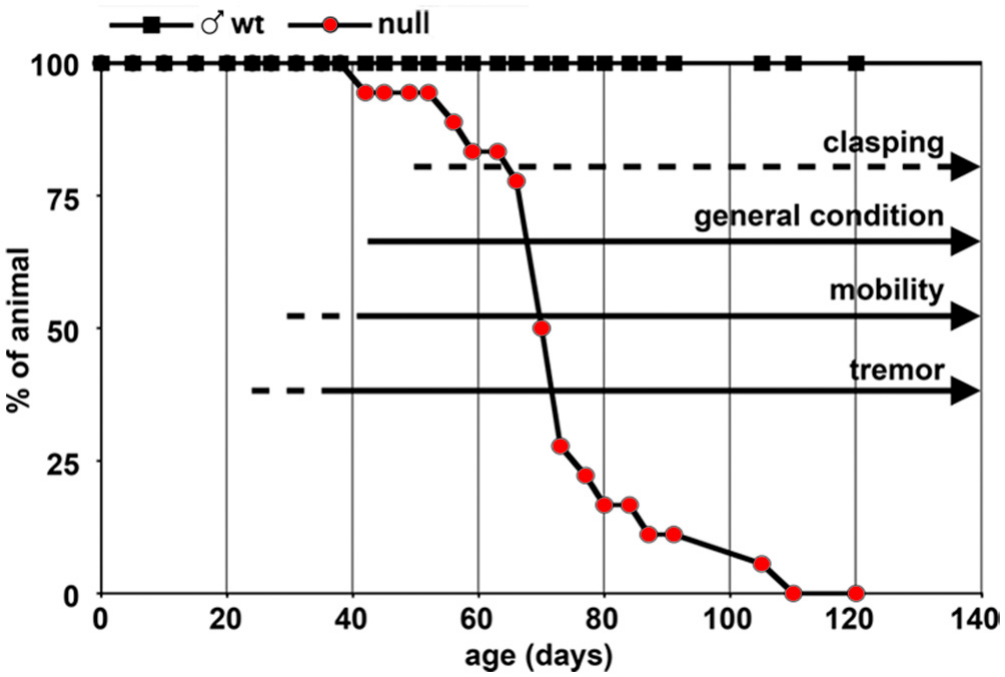
CD1 *Mecp2*-null animals have a life span comparable to that of B6 null animals. CD1 null animals (red circles) are compared to wt controls (black squares). Arrows represent the timing of appearance of the different symptoms described in Fig 2 (dotted lines = 50–99% of animals have the given symptom, continuous line = 100% of the animals have the symptom; n = 18 *Mecp2*-null).

Uneven wearing of teeth and misalignment of the jaws are fairly usual in B6 null animals [10], while they were never noticed in CD1 null animals. After 6–7 weeks of age, CD1 null animals are easily distinguishable from their wt littermates as they show ungroomed coat and hunched stance. The worsening of the general conditions immediately precedes death and mortality, usually peaks between P70 and P80 (Fig 3). Animals are often euthanized at this age for ethical reasons due to the presence of severe symptoms, but given the variability in the severity of symptoms, some animals survive even longer. In rare cases animals become strongly symptomatic and suddenly die even at earlier ages. In the case of *Mecp2*^+/-^ females, the worsening of the animal conditions does not lead to early natural death.

**Fig 4:**
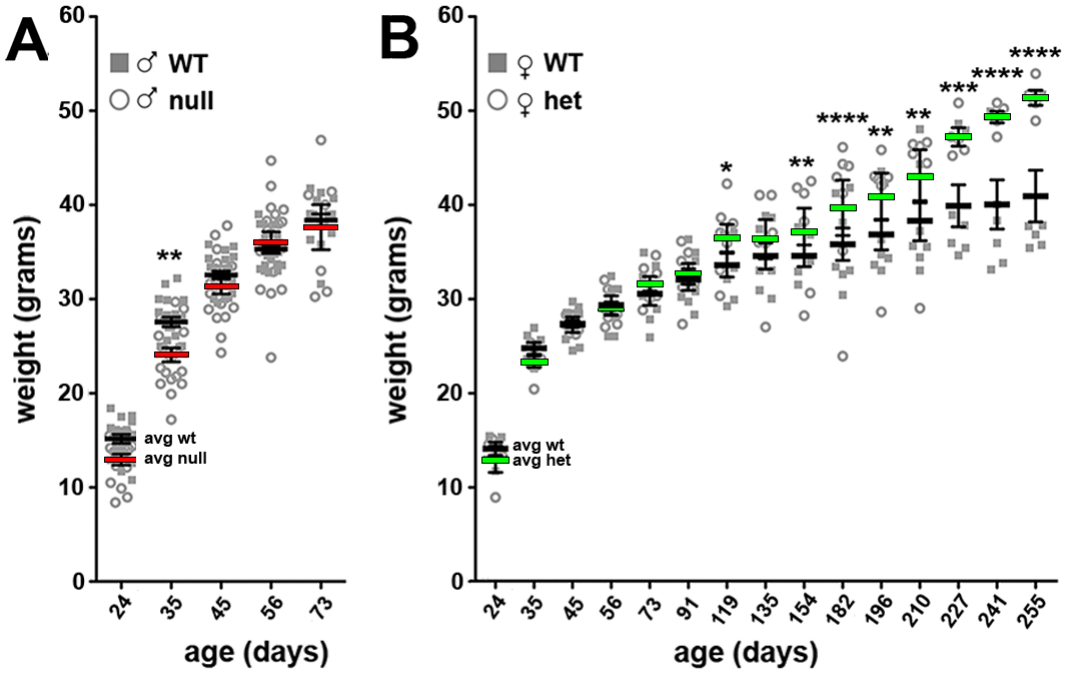
The weight of CD1 null mice is mildly reduced compared to wt while it is altered in heterozygous females. **A**: Young CD1 null mice display a subtle reduction of body weight compared to wt animals. The weight reaches wt levels at around 50 days of age. **B**: Beginning from three months of age *Mecp2*^*+/-*^ animals result overweighed compared to wt controls. Importantly, Mecp2^*+/-*^ females do not die spontaneously, thus the data are based on the day of euthanasia. Statistical significance was evaluated through two-way ANOVA, Bonferroni’s post-hoc test: * = p<0,05; ** = p<0,01; *** = p<0,001; **** = p<0,0001; n = 21 wt males, 20 null, 7 *Mecp2*^*+/-*^ and 7 wt females.

In general, CD1 and B6 mice have different body size, with CD1 animals weighing significantly more compared to B6 mice (the average adult CD1 weight is 40 gr, while adult B6 mice weigh 20 gr). For this reason, handling CD1 animals might be easier in some procedures compared to B6 mice. At P35, the weight of CD1 *Mecp2*-null animals is moderately (although significantly) reduced compared to controls but reached wt levels after roughly 6–7 weeks of age (Fig 4A); on the contrary, B6 null animals are strongly underweighted compared to wt mice ever since birth [10,23]. Notably, in rare cases a rapid decrease in weight is observed in the CD1 model. *Mecp2* heterozygous females do not show any particular difference in weight compared to wt until 3 months of age, when a progressive increase is observed (Fig 4B).

**Fig 5:**
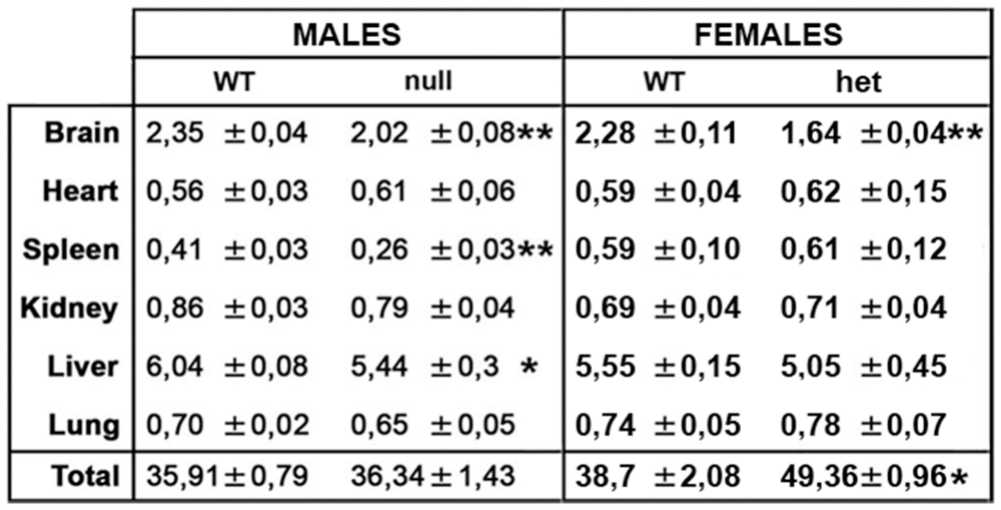
*Mecp2*-null and heterozygous mice display a reduction in the size of specific organs. The weight of CD1 null organs compared to wt males resulted reduced for brain, spleen and liver. *Mecp2*^*+/-*^ females displayed a reduced brain size and an increase in overall size at the time of sacrifice (the weight of the organs is represented as percentage of the total body weight of each animal). Statistical significance was evaluated through Student’s t-test; * = p<0,05; ** = p<0,01; n = 12 wt males, 10 null, 8 *Mecp2*^*+/-*^ and 9 wt females.

As previously found in B6 *Mecp2*-null and heterozygous animals and in accordance with RTT patients, a macroscopic evaluation of organs dissected from symptomatic animals (both males and females) highlighted a decrease in brain weight [11, 23, 24, 25]. Interestingly, spleen and liver weights (Fig 5) are reduced in males but not in females.

**Fig 6:**
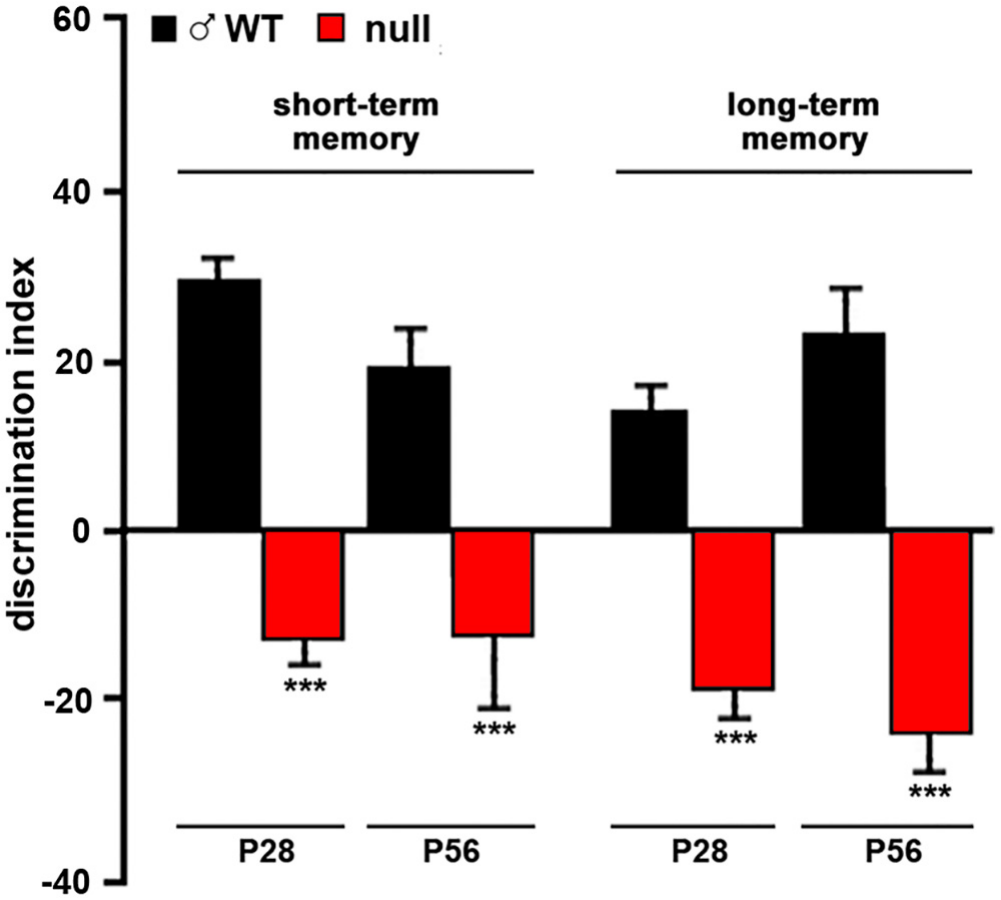
Cognition of CD1 *Mecp2*-null animals is impaired. Cognition of CD1 wt and null animals was tested with the novel object recognition test. Animals were assayed at P28 (30 wt and 27 null) and P56 (17 wt and 10 null) and displayed strong impairments of both short and long term memory at both ages. Statistical significance was evaluated through Student’s t-test; *** = p<0,001.

Although the severity of cognitive alterations is still debated in RTT, many studies demonstrate that mouse models of *Mecp2* diseases are characterized by severe cognitive impairments. No cognitive data are available for the B6 model; however, reduced freezing time during cued fear conditioning tests and diminished time spent exploring a novel object (NOR: Novel Object Recognition test) affect a variety of other RTT mouse models (*Mecp2*^*tm1*.*1jae*/Y^, *Mecp2*^308^, *Mecp2*^R168X^, *Mecp2*^S421A^, *Mecp2*^T158A^; [26–31]). To assess the presence of cognitive deficits in short- and long-term memory, CD1 wt and *Mecp2*-null mice were tested with NOR at different ages (Fig 6). *Mecp2*-null males showed a significant impairment in short-term memory with respect to wt already at P28, as stated by the significant reduction of the discrimination index (144%, t = 8,978; p<0,0001). A greater cognitive impairment was observed when long-term memory was tested. Indeed, the discrimination index was significantly reduced compared to wt (235%, t = 6,381; p<0,0001). The cognitive deficit persisted also at P56, when the neurological symptoms in *Mecp2-null* animals are fully manifested. In fact, the discrimination index was significantly reduced in short- and long-memory by 168% (t = 3.860; p = 0.0007) and 203% (t = 5.940; p<0.0001), respectively.

Next, we investigated whether some typical morphological and molecular alterations displayed by different *Mecp2*-null mice are present also in the CD1 model.

**Fig 7:**
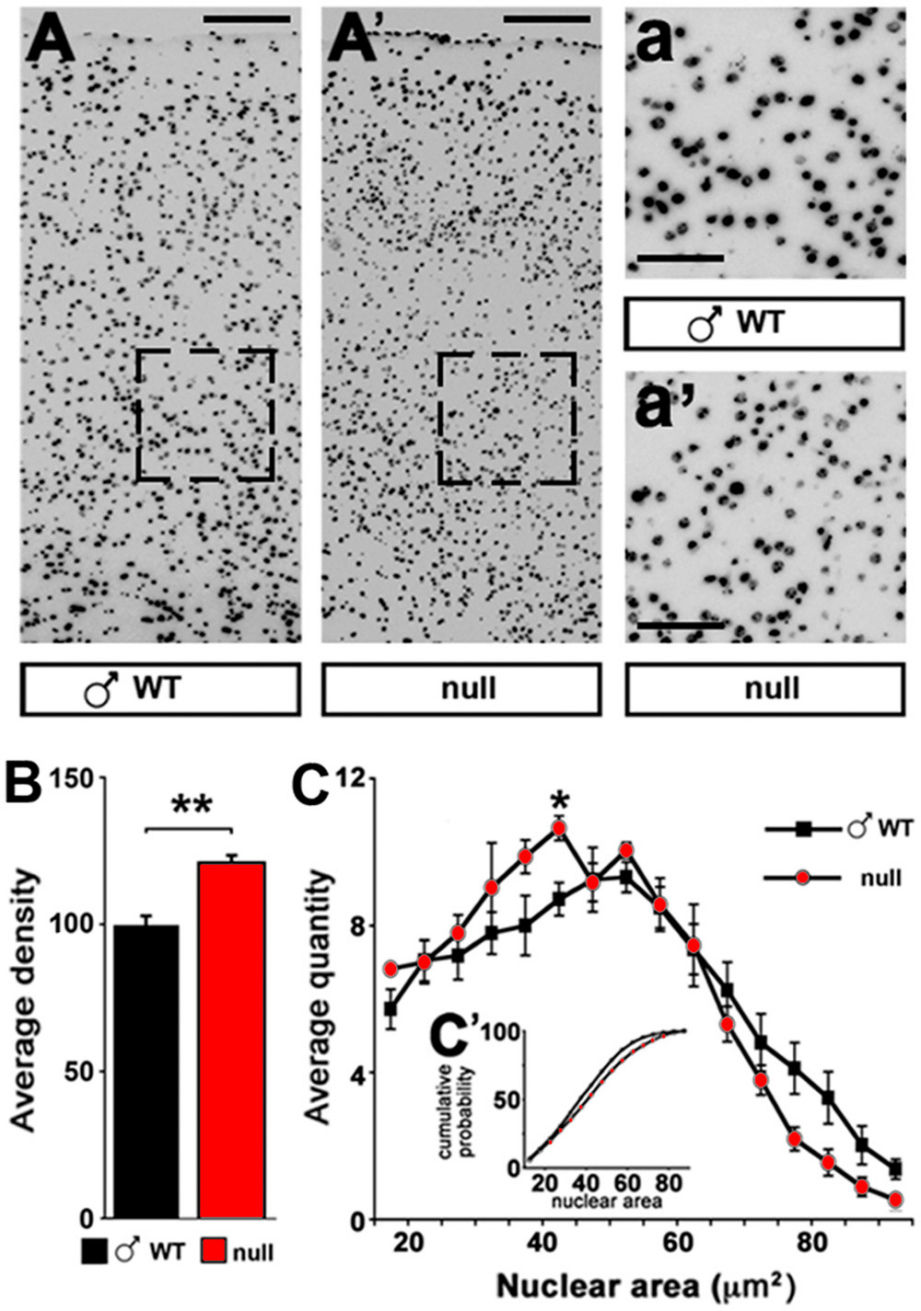
Morphological defects featured by CD1 *Mecp2*-null cortices. **A, A’**: Representation of nuclear staining (DAPI) in wt and *Mecp2*-null frontal cortical lobes. **B**: Cell density is increased in *Mecp2*-null cortices compared to wt samples. Statistical significance was evaluated through Student’s t-test ** = p<0,01; n = 6 wt and 4 null at P60. **C**: Nuclear size is reduced in *Mecp2*-null samples stained with DAPI. **C’**: Kolmogorov-Smirnov test revealed a maximum distance between the two groups of 7,7%, with a p<0,01; * = p<0,05 post-hoc Student’s t-test; n = 6 wt and 4 null at P60. (Scale bar: A, A’ 100 μm; a, a’ 50μm).

**Fig 8:**
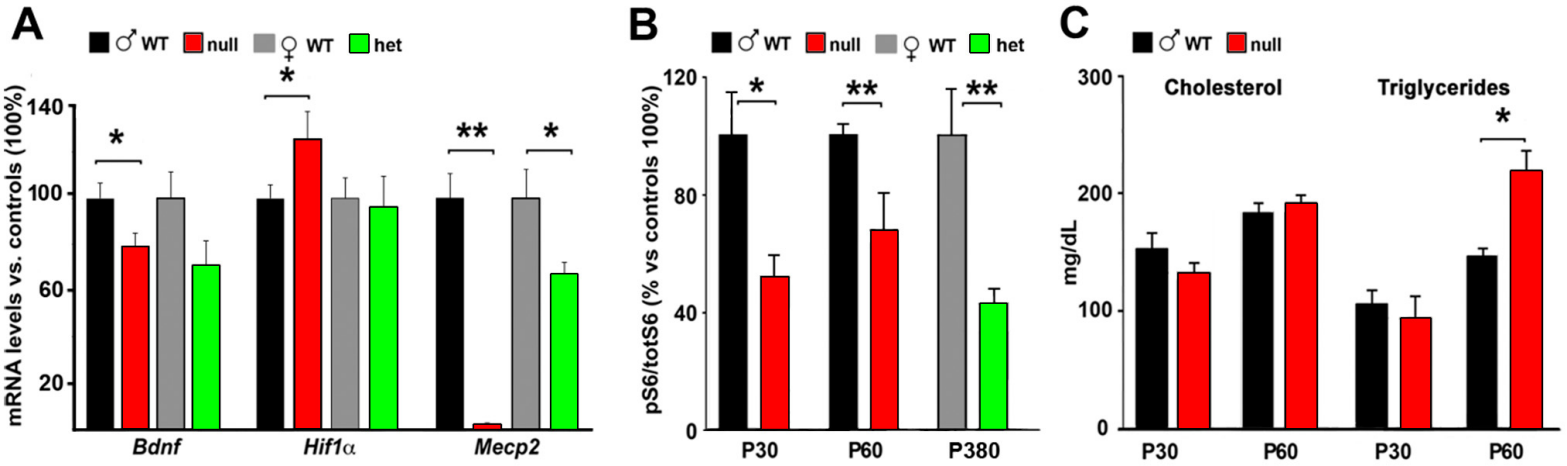
CD1 *Mecp2*-null animals largely reproduce previously described molecular phenotypes. **A**: qPCRs comparing the expression of different genes (BDNF, Hif1α, *Mecp2*) in CD1 wt or null males (P60), and wt or heterozygous females (P≥240). Data are expressed as percentage of null or heterozygous samples versus wt male or female controls (100% ±SEM). (Student’s t-test; * = p<0,05; ** = p<0,01; n = 9 null, 10 wt males, 8 het and 9 wt females). **B**: Western blots representing the down-regulation of rpS6 phosphorylation (on serine 240–244) in CD1 null animals at P30 and P60 in males (n = 3 wt and 4 null at P30; n = 5 null and 7 wt at P60) and in *Mecp2*^*+/-*^ females at P≥240 (n = 7 wt and 7 heterozygous). Statistical significance was evaluated through Student’s t-test; * = p<0,05; ** = p<0,01; **C**: Serum cholesterol and triglycerides levels in slightly and fully symptomatic animals. Statistical significance was evaluated through Student’s t-test; * = p<0,05; n = 4 wt and 6 null at P30; n = 8 wt and 9 null at P60.

As expected [32], cortical neurons of symptomatic CD1 *Mecp2*-null mice are more densely packed (Fig 7A, 7A’ and 7B) and exhibit smaller nuclei with respect to wt controls (Fig 7C). The cerebral cortex of CD1 null animals confirms a reduced transcription of the plasticity modulator *Bdnf* [33]. In accordance with previous observations made on the B6 *Mecp2*-null model [34], we also demonstrate a significant up-regulation of *Hif1*α transcription in the CD1 null cortex (Fig 8A). The deregulation of this transcript might be due to the reduction of oxygen levels in the blood related to a generalized status of chronic hypoxia, in line with the common feature of RTT patients suffering from respiratory abnormalities. As expected, in heterozygous females the levels of transcription of *Mecp2* is reduced of roughly 40%; in these animals we detected a similar (although not significant) tendency to the reduction in the levels of *Bdnf*, but we did not detect any defect in *Hif1*α expression. Moreover, reduced AKT/mTOR signalling, originally described in the B6 Jaenisch null animals [35] and then confirmed in other cellular and animal models of *Mecp2* [23,36], is evident also in the CD1 background. The level of rpS6 phosphorylation is in fact significantly reduced in both young (P30) and old (P60) *Mecp2*-null animals and in old symptomatic (P≥240) heterozygous females (Fig 8B).

It is known that RTT patients display different metabolic alterations [30]; these impairments are reproduced in mouse models of the disease depending on their genetic background [13,21,37,38,39]. Accordingly, the metabolism of peripheral cholesterol (total serum cholesterol, low-density lipoprotein and triglyceride) is abnormally elevated in the 129*Mecp2*^tm1.1Bird/Y^ mouse model at P60, but not in the B6*Mecp2*^*tm1*.*1jae*/Y^ [34]. *Mecp2*^tm1.1Bird/Y^ mice maintained on a mixed 129S6B6F1 background reveal markedly increased triglycerides levels throughout life and increased cholesterol after 6 weeks of age [13], whereas the *Mecp2*^*tm1*.*1jae*/Y^ line kept on a mixed 129SvEV-C57BL6-BALB/c background shows increased serum cholesterol and triglycerides at 7 weeks of age [40]. Our CD1 *Mecp2*^tm1.1Bird/Y^ model show unaltered levels of cholesterol both in pre- and post-symptomatic stages, but, in line with previous observations, triglycerides levels are increased at P60 once the symptoms became evident (Fig 8C). Overall these data seem to suggest that cholesterol levels are more affected than those of triglycerides by modifiers gene and, consequently, by the genetic background. Therefore, in *Mecp2*-null mice metabolic phenotypes strongly depend on the genetic background whereas neurological alterations seem to be less sensitive to genetic modifiers.

## Conclusions

Mutations in *MECP2* cause a broad spectrum of neurological disorders generally characterized by cognitive deficiencies, motor impairments and seizures. Neurodegeneration is not observed in mice mimicking MeCP2 disorders or in RTT patients, and phenotypic rescue is possible upon *Mecp2* re-activation in animal models [6–9], suggesting that successful therapeutic intervention are possible. However, no effective treatment for RTT syndrome and *MECP2*-associated disorders currently exists. The major challenge in the field is therefore the identification of molecular pathways associated with the pathology that can be targeted with specific treatments. Such treatments obviously need thorough testing in mouse models of *Mecp2* alterations, to assess their effectiveness in ameliorating the general health condition or specific clinical symptoms [36]. Because of that and their vast use in the field, *Mecp2*-null mouse models hold great promise for translational research. However, the poor breeding capabilities, the reduced litter size and the bad health conditions of transgenic animals can represent a serious limiting factor.

It is well known that the phenotype developed by *Mecp2*-deficient animals can vary according to the genetic background and, therefore, to its specific set of modifier genes. Previous publications [13,40] and our present study suggest that this is particular true for metabolic alterations. Therefore, promising treatments should be tested on more than one mouse model/strain of *Mecp2* [41]. A detailed description of the different features displayed by *Mecp2* strains harbouring the same genetic mutation would thus aid investigators in choosing the proper genetic background for their studies [20].

Here, we demonstrate that, compared to the B6 strain, the novel CD1 *Mecp2*^tm1.1Bird^ strain presents a higher breeding success and larger litters that would facilitate basic and translational studies, including heterozygous mice and pregnant females. In our experience these advantages have so much accelerated our studies, while reducing the number of housed animals and therefore the costs, to overwhelm the disadvantage of using an outbred strain that is generally characterized by increased variability. Moreover, it is worthwhile to remind that because the molecular defects produced by lack of MeCP2 are often mild in magnitude [5], a high number of animals is anyhow often necessary to obtain significant data.

The CD1 hemizygous *Mecp2*-null male recapitulates most of the measurable outcomes described in the B6 background both at the behavioural, cellular and molecular level. Accordingly, this model was already used to highlight maturation defects driven by lack of Mecp2 during both embryonic [42] and adult [43] development. In conclusion, we suggest that this novel mouse strain has a high potential to accelerate and integrate research on Rett syndrome and *MECP2*-related pathologies and should be considered as a valid tool for future basic and/or translational studies.
